# Spatiotemporal Regulation of Circular RNA Expression during Liver Development of Chinese Indigenous Ningxiang Pigs

**DOI:** 10.3390/genes13050746

**Published:** 2022-04-24

**Authors:** Wenwu Chen, Haiming Ma, Biao Li, Fang Yang, Yu Xiao, Yan Gong, Zhi Li, Ting Li, Qinghua Zeng, Kang Xu, Yehui Duan

**Affiliations:** 1 College of Animal Science and Technology, Hunan Agricultural University, Changsha 410128, China; cww1242646778@163.com (W.C.); y621829@163.com (F.Y.); xiaoyu1030189228@126.com (Y.X.); 13910008175@139.com (Y.G.); zhili963@foxmail.com (Z.L.); gaohu_20190008@163.com (T.L.); cws751840156@163.com (Q.Z.); 2 Guangdong Laboratory for Lingnan Modern Agriculture, Guangzhou 510642, China; 3 College of Animal and Veterinary Sciences, Southwest Minzu University, Chengdu 610000, China; 4 Laboratory of Animal Nutritional Physiology and Metabolic Process, Institute of Subtropical Agriculture, Chinese Academy of Sciences, Changsha 410125, China; 5 Ningxiang Pig Farm of Dalong Livestock Technology Co., Ltd., Ningxiang 410600, China; xukang2020@163.com (K.X.); duanyehui@isa.ac.cn (Y.D.)

**Keywords:** RNA-Seq, ceRNA, STEM, lipid, RT-qPCR

## Abstract

Background: There have been many studies on the relationship between circRNAs and fat deposition. Although the liver is a central organ for fat metabolism, there are few reports on the relationship between circRNAs in the liver and fat deposition. Methods: In this study, we systematically analyzed circular RNAs in the liver of Ningxiang pigs, at four time points after birth (30 days, 90 days, 150 days and 210 days). Results: A total of 3705 circRNAs were coexpressed in four time periods were found, and KEGG analysis showed that the significantly upregulated pathways were mainly enriched in lipid metabolism and amino acid metabolism, while significantly downregulated pathways were mainly related to signal transduction, such as ECM–receptor interaction, MAPK signaling pathway, etc. Short time-series expression miner (STEM) analysis showed multiple model spectra that were significantly enriched over time in the liver. By constructing a competing endogenous RNA (ceRNA) regulatory network, 9187 pairs of networks related to the change in development time were screened. Conclusions: The expression profiles of circRNAs in Ningxiang pig liver were revealed at different development periods, and it was determined that there is differential coexpression. Through enrichment analysis of these circRNAs, it was revealed that host genes were involved in metabolism-related signaling pathways and fatty acid anabolism. Through STEM analysis, many circRNAs involved in fat metabolism, transport, and deposition pathways were screened, and the first circRNA–miRNA–mRNA regulation network map in Ningxiang pig liver was constructed. The highly expressed circRNAs related to fat deposition were verified and were consistent with RNA-Seq results.

## 1. Introduction

As a famous local pig breed in China, the Ningxiang pig has the characteristics of tender meat, high intramuscular fat content, and strong fat deposition ability [1]. With the increasing demand for quality pork products, Ningxiang pork is popular among Chinese consumers because of its good taste and high nutritional value. Therefore, in recent years, there has been a large number of research reports related to Ningxiang pig [2]. For example, He found that, in Ningxiang pig, monounsaturated and polyunsaturated fatty acids account for 43.10% and 12.82%, respectively; arachidonic acid (AA; C20:4n6) and docosahexaenoic acid (DHA; C22:6n3) account for 2.29% and 0.14% of total fatty acids in the longissimus dorsi muscle of Ningxiang pig, which is much higher than Duroc [3].

As an important metabolic organ in mammals, the liver is the central part of carbohydrate storage, but also an important part of lipid metabolism. For example, in mammals, the synthesis sites of triacylglycerol, phospholipids, and fatty acids are mainly liver and adipose tissue, and the enzyme system extending the carbon chain of fatty acids also exists in the endoplasmic reticulum of hepatocytes. Therefore, the synthesis of fatty acids is inseparable from the regulation of various molecules in the liver [4].

The regulation of the liver on adipose tissue is a complex process, in which there are a series of small molecule interventions. miRNA is an endogenous noncoding small RNA with a length of 22nt, which has the function of regulating gene expression. Studies show that miRNA plays a crucial role in the process of liver regulation of adipose tissue. For example, Ahn [5] found that the downregulation of miR-467b leads to the upregulation of lipoprotein lipase (LPL)in liver tissue. Additionally, the main function of LPL is to increase the volume of adipocytes and promote adipocyte differentiation [6].

In recent years, with the development of bioinformatics and the progress of sequencing technology, circRNAs are identified in many species. circRNA, as an RNA molecule with a circular structure formed by reverse splicing, is of great significance for the regulation of gene expression. Accumulating evidence shows that circRNAs regulate a diversity of cellular processes by acting as miRNA sponges, anchors for circRNA binding proteins (cRBPs), transcriptional regulators, molecular scaffolds, and sources for translation of small proteins/peptides. It is reported that circRNA participates in the process of fat deposition and fat metabolism and promotes fat deposition through the sponge adsorption of miRNA [7,8,9].

Although there are many reports on the regulation of adipogenesis by circRNA in the liver in the human field [10,11], there are few reports on pigs. This study uses RNA-sequencing (RNA-Seq) technology to systematically identify the circRNAs and miRNAs of the liver at four developmental stages (30, 90, 150, and 210 days after birth). The purpose is to provide a molecular basis for the follow-up study of Ningxiang pigs and also to provide candidate genes for circRNA function research. At the same time, the discovery of this study fills the gap in the molecular mechanism of the relationship between porcine fat deposition and liver circRNA.

## 2. Materials and Methods

### 2.1. Animals and Sample Collection

In this study, 12 male purebred Ningxiang pigs (all half-sib pigs, Ningxiang pig farm of Hunan Dalong animal husbandry technology Co., Ltd., Changsha, China) were selected. Liver samples were collected at 30d (30 days old), 90d (90 days old), 150d (150 days old), and 210d (210 days old) in four development stages. Liver samples at each stage were biologically repeated three times. They were placed under standard environmental conditions (including uncontrolled room temperature and natural light), fed a standard diet three times, and drank freely every day. Then, according to the standard procedure, three healthy Ningxiang pigs with similar weights were randomly selected from four age groups. Pigs fasted the day before slaughter. After that, euthanasia was performed by bleeding under the surgical plane of isoflurane (4.5% tidal volume mask) anesthesia. The experimental animals were the same as those used to study the muscle and adipomics of Ningxiang pigs [12]. Liver samples were collected within 30 min after slaughter, immediately frozen in liquid nitrogen, and stored at −80 °C until analysis. All animal experiments in this study were approved by the Institutional Animal Care and Use Committee of Hunan Agricultural University, Changsha, Hunan Province, China under approval number 2013-06. 

### 2.2. RNA Extraction

Total RNA was extracted according to the manufacturer’s protocol; briefly, an animal RNA purification reagent was used to obtain RNA from tissues, and genomic DNA was removed by rDNA ribonuclease (Takara bio Inc., Dalian, China). Afterward, the RNA quality was verified by using a 2100 biological analyzer (Agilent technology, Santa Clara, CA, USA) and ND-2000 spectrophotometer (Waltham Seymour Fisher Technology, Waltham, MA, USA). The screening and determination results were OD260/280 = 1.8~2.2, OD260/230 ≥ 2.0, RIN ≥ 6.5, 28S:18S ≥ 1.0, >10 μg high quality RNA. The RNA sample set was used to construct the sequencing library.

### 2.3. Library Preparation and Sequencing

The RNA-Seq transcriptome strand library was constructed according to the TruSeq^TM^ Stranded Total RNA kit from Illumina (San Diego, CA, USA), which was prepared with 5 μg of total RNA. The first cDNA strand was synthesized by using an IlluminaRibo-Zero Magnetic kit ribosomal RNA (rRNA) depletion with random hexamer primers. Then, the RNA template was removed, the replacement strand was synthesized, and dUTP was replaced with dTTP to generate ds cDNA. Using AMPure XP beads to separate ds cDNA from the reaction mixture. When a single “A” nucleotide was added to the 3′ end, multiple index adapters would also be connected to the ends of ds DNA. The cDNA size was selected on 2% UltraAgarose, and then PCR amplification was performed using Phusion DNA polymerase (NEB). After quantification by TBS380, the paired-end RNA sequencing library was sequenced with Illumina HiSeq 4000. In addition, 3 μg of total RNA was connected to the sequencing adapter using TruSeq^TM^ Small RNA Sample Prep Kit (Illumina, San Diego, CA, USA). Subsequently, cDNA was synthesized by reverse transcription and a library was generated. Finally, using the library, we conducted deep sequencing at Shanghai Meiji Biomedical Biotechnology Co., Ltd. (Shanghai, China).

### 2.4. Read Mapping and Transcriptome Assembly

The SeqPrep (https://github.com/jstjohn/SeqPrep, accessed on 2 January 2021) was used with default parameters to trim and quality control the raw paired-end reads. Then, the clean reading was aligned with the reference genome (accession number PPJNA531381) using HISAT2 (https://ccb.jhu.edu/software/hisat2/index.shtml, accessed on 5 January 2021) software. The mapped reads of each sample were assembled by StringTie (https://ccb.jhu.edu/software/stringtie/index.shtml, accessed on 20 January 2021) using a reference-based method.

### 2.5. Identification of circRNAs

The CircRNA Identifier 2 (CIRI2) was used in conjunction with find_circ to identify circRNA. The SAM file was scanned twice, and information was collected to identify and characterize circRNA. Then, the identified circRNAs were output, together with the annotation information. The expression level of each circRNA was calculated according to the reads per million mapped reads (RPM) method.

### 2.6. Differential Expression Analysis and Functional Enrichment

Deseq2 was used to extract the significantly differentially expressed (DE) circRNAs with |log2FC| > 1 and adjusted *p*-value ≤ 0.05. Functional enrichment analysis and KEGG enrichment analysis were used to determine whether Dec was significantly enriched in those metabolic pathways at *p*-values ≤ 0.05, compared with the whole transcriptome. KEGG pathway analysis was performed using KOBAS.

### 2.7. Time-Series Analysis

Time-series analysis was performed by the short time-series expression miner (STEM) method [13]. STEM is a tool that can find genes with specific expression patterns. After importing the data of four different stages of the liver into STEM software, we can rely on the powerful computing power of stem to cluster the genes with the same expression trend to obtain clusters. Each cluster shows a group of genes with the same expression trend. In the results, clusters with gray backgrounds showed no significant trend, while those with color backgrounds showed significant trends (*p* < 0.05). Clusters with the same color showed similar gene expression trends. Significantly enriched model profiles are indicated by different colors (Benjamini–Hochberg-adjusted *p*-values ≤ 0.05). The corrected *p*-values are sorted from small to large.

### 2.8. Analysis of ceRNAs Regulatory Networks

To reveal the role and interactions among ncRNAs and mRNAs, we constructed an ncRNA–mRNA regulatory network. MiRanda was used to predict the circRNA–miRNA–mRNA pairs (score cutoff ≥ 160 and energy cutoff ≤ −20). The coexpression network of circRNA–miRNA–mRNA was constructed using Cytoscape software (v3.2.1) to investigate the function of key circRNAs [14].

### 2.9. Validation of Expression by PCR

An RNA-easy Isolation Reagent (Vazyme, Nanjing, China) was used to extract RNA based on the manufacturer’s protocol. Real-time quantitative reverse transcription PCR (RT-qPCR) was performed using a cDNA Synthesis Kit (Vazyme) and qPCR Probe Kit (Vazyme). Relative mRNA expression was analyzed with the 2^−ΔΔCT^ method and standardized to those of the appropriate internal references. Divergent primers were used for PCR amplification of reverse-transcribed cDNA, and 2% agarose gel was selected for agarose gel electrophoresis [15].

### 2.10. Statistical Analysis

DESeq2, which is a method of differential analysis of count data, was used in the differential gene expression analysis of negative binomial distribution. To improve the stability and interpretability of the estimation, shrinkage estimation was used to estimate the dispersion and fold changes. This allows a more quantitative analysis to focus on intensity, not just the existence of differential expression. In DESeq2, the Wald test is usually used for hypothesis testing when comparing two groups. We used the R script to perform a KEGG PATHWAY enrichment analysis on the genes in the gene set. When the *p* value < 0.05, the KEGG PATHWAY function was considered to be significantly enriched. To determine the occurrence of differential expression of all detected genes, expression level difference analysis was used, which is a statistical inference process. Due to the large number of genes involved, statistical tests had to be performed multiple times. We corrected the *p*-value obtained from statistical tests several times to ensure that the false discovery rate was controlled. Then, the adjusted *p*-value smaller than 0.05 was significant.

## 3. Results

### 3.1. Identification and Characteristics of circRNAs in Ningxiang Pig Liver

**Figure 1:**
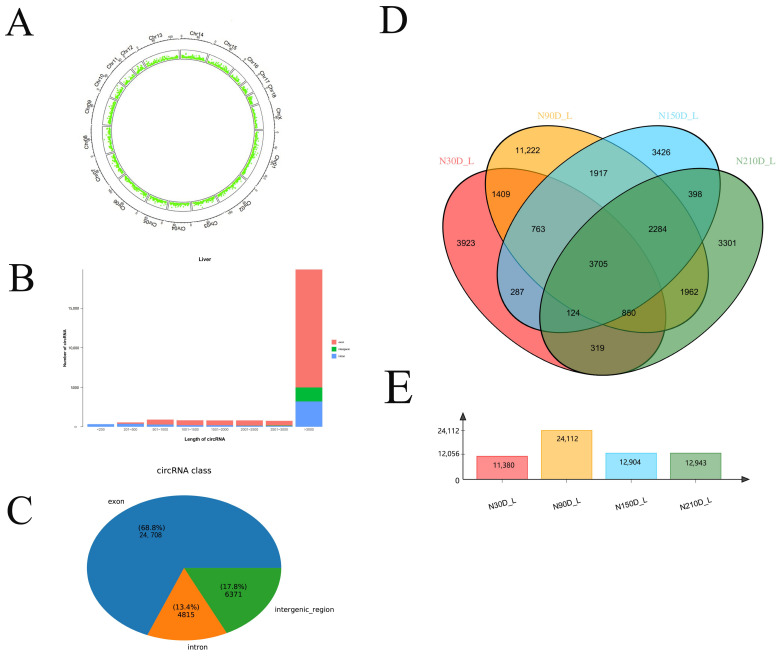
Identification and feature of circRNAs in Ningxiang pig: (**A**) distribution of circRNAs in each chromosome; (**B**) classification and length distribution of circRNAs in Ningxiang pig liver; (**C**) Venn diagram of circRNAs identified at four development points in liver of Ningxiang pigs; (**D**) Venn diagram of circRNAs identified at four development points in liver of Ningxiang pigs; (**E**) Number of circRNAs identified at four development points in liver of Ningxiang pigs.

Using Illumina HiSeqxten sequencing, we obtained 56,500,489, 55,531,984, 45,030,983 and 51,897,636 raw reads from the four developmental stages (30, 90, 150, and 210 days after birth) of liver, respectively (Table 1). In this study, after low-quality and adaptor-polluted reads were first removed from the raw data, the Q30 value of the clean reads of the liver at 30, 90, 150, and 210 days was greater than 95%. Among the samples mapped with the Ningxiang pig genome (accession number PPJNA531381) assembled by our research group, the mapping rate of each sample exceeded 92%. These mapped sequences were used for subsequent circRNA identification (Supplemental Table S1). Analysis of chromosome distribution indicated that the identified circRNAs were transcribed widely and unevenly on the chromosome (Supplemental Table S2). Compared with other chromosomes, most of the circRNAs identified were distributed on chromosome 1, chromosome 6, and chromosome 13, which is consistent with the characteristics of circRNA reported by others. In addition, we also detected circRNA in sex chromosomes (Figure 1A). As shown in previous studies, most circRNAs contain multiple exons, and some circRNAs also retain introns. The length analysis showed that most circRNAs were longer than 3000 bp (Figure 1B). In this study, most of the circRNAs identified (68.8%) were exons, followed by intergenic (17.8%) circRNAs, and only a small portion (13.4%) were located in introns (Figure 1C). We further constructed Venn maps of the identified circRNAs in the liver and at four time points (Figure 1D). At the same time, it was found that a total of 3705 circRNAs were coexpressed in four time periods, whereas the number of circRNAs identified at 90d was the largest, and the number of circRNAs that were uniquely expressed at 90d was far more than other time points (Figure 1E). circRNA also showed time-specific expression in the liver during Ningxiang pig development.

### 3.2. Spatiotemporal Dynamic Expression Pattern of circRNAs in Liver of Ningxiang Pigs

To understand the regulation of circRNAs in the liver, we performed expression profiling of circRNAs in the liver at postnatal days 30, 90, 150, and 210. This profiling allowed the evaluation of dynamic changes in liver circRNA expression from lactation to fattening and screening out circRNAs related to fatty acid anabolism and transport.

**Figure 2:**
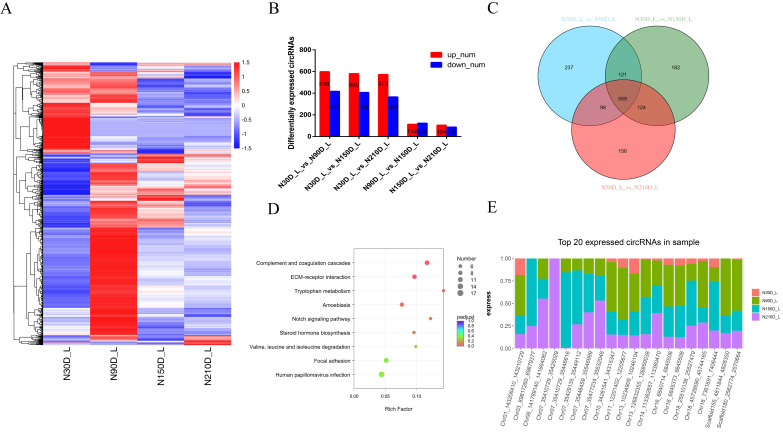
Differentially expressed (DE) circRNAs and their expression modes in liver: (**A**) heat map of all DE circRNAs among the five compared groups (30d vs. 90d, 30d vs. 150d, 30d vs. 210d, 90d vs. 150d, and 150d vs. 210d groups); (**B**) number of differentially expressed circRNAs in liver vs. means versus; (**C**) the number of common DE circRNAs in liver; (**D**) KEGG pathway analysis of common DE genes in liver. The top 10 enriched KEGG pathways ranked by *p*-values are shown; (**E**) top circRNAs expressed in liver.

We clustered the 3362 differential circRNAs identified in the five comparison groups (Figure 2A and Supplemental Table S3). Between two closed time points, we detected 598 circRNAs upregulated at 90d, 580 circRNAs upregulated at 150d, and 572 circRNAs upregulated at 210d, compared with 30d (Figure 2B and Supplemental Table S4). KEGG analysis showed that, between two closed time points in liver tissue, the significantly upregulated pathways were mainly enriched in lipid metabolism and amino acid metabolism such as tryptophan metabolism, primary bile acid biosynthesis, valine, leucine, and isoleucine degradation, Retinol metabolism, etc; inversely, the significantly downregulated pathways were mainly related to signal transduction, such as Notch signaling pathway, ECM–receptor interaction, MAPK signaling pathway, etc (*p* < 0.05 and Supplemental Table S5).

Compared with 30d, 559 circRNAs were identified as common DE genes during the entire liver development process (Figure 2C). KEGG pathway analysis also found that these common DE genes were mainly enriched in complement and coagulation cascades, ECM–receptor interaction, tryptophan metabolism, Notch signaling pathway, steroid hormone biosynthesis, etc. (Figure 2D). Next, we observed that several of the most abundant circRNAs in the liver originated from protein-coding genes with pivotal roles in lipid synthesis and metabolism (e.g., Scaffold180_2562774_2570664, Scaffold155_4811844_4828350, and Chr01_143206410_143210729) (Figure 2E and Supplemental Table S6).

### 3.3. Constructing the circRNA–miRNA–mRNA Coexpression Networks through Time-Series Analysis

**Figure 3:**
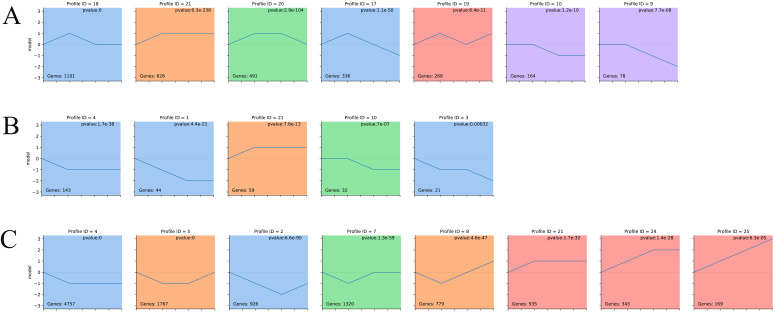
Time-series modules of circRNAs: (**A**) time-series modules of circRNAs in liver; (**B**) time-series modules of miRNAs in liver; (**C**) time-series modules of mRNAs in liver. Numbers on the top indicate module numbers. Numbers on the top right corner indicate the statistically significant *p*-value. Numbers in the lower-left corner indicate the numbers of circRNAs in each module. The colored profiles are indicated by different colors (Bonferroni-adjusted *p*-values ≤ 0.05). The *p*-value is sorted from small to large. If the profile is the same color, it means that these profiles belong to the same cluster.

The analysis results which dynamic expression patterns across the four development stages we found that all the identified circRNAs were classified into five cluster profiles in the liver, containing seven enriched model profiles (Figure 3A and Supplemental Table S7). Gradual increases and decreases were distributed in profiles 21, 10, and 9, while biphasic responding expression patterns occurred in module profiles 18, 20, and 17. Finally, we only considered the colored and largest modules. By using the functional enrichment analysis, we found that the largest modules in the liver are enriched in a variety of biological processes such as transport and catabolism, cell growth and death, amino acid metabolism, lipid metabolism, etc. (Supplemental Table S8).

**Figure 4:**
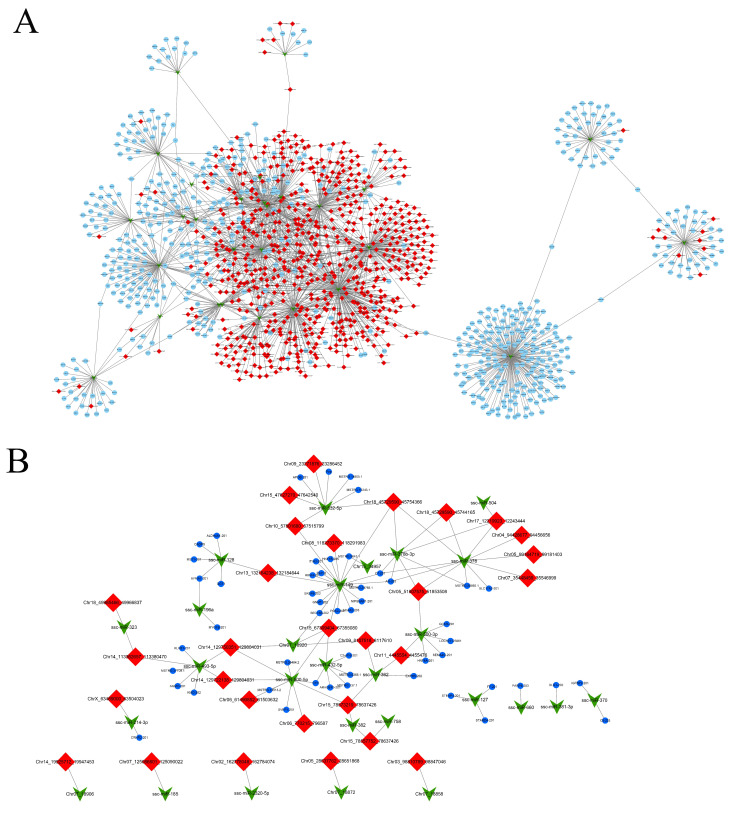
A view of interaction among circRNAs–miRNAs–mRNAs in liver: (**A**) mRNAs were associated with miRNA profile 4 and circRNA profile 21 to obtain the ceRNA regulation network; (**B**) red diamond nodes represent circRNAs; blue circle nodes represent mRNAs; green arrow nodes represent miRNAs.

According to previous relevant studies, sponge adsorption exists between circRNA and miRNA, while the miRNA regulates the function of mRNA. In order to further understand the relationship between circRNA, miRNA, and mRNA, we also performed liver STEM analysis for miRNA and mRNA (Figure 3B,C). Based on those results, we found that all the identified miRNAs were classified into three cluster profiles and five enriched model profiles, whereas mRNAs were classified into four cluster profiles and eight enriched model profiles. There were similar expression patterns in model profile 21 of circRNA, miRNA. and mRNA, which suggests a high correlation during different stages. To further explore the circRNA–miRNA–mRNA relationship, we selected circRNAs in the colored modules to predict the miRNA and used the intersection with the miRNAs that were significant in the miRNA STEM analysis; then, target analysis was performed with mRNAs that were significant in mRNA STEM analysis. There were 9187 network pairs, which related to developmental time changes screened out (Figure 4A). Through further screening, we found that there are important marker genes related to lipid metabolism in this ceRNA network, such as ACSL3, ACSL5, APOA4, FADS, etc. (Figure 4A and Supplemental Table S9).

There was a competing relationship between the circRNA and miRNA, and at the same time, the mRNA and miRNA could bind; the expression trend of circRNA was similar to that of mRNA but opposite to that of miRNA. circRNA, miRNA, and mRNA were compared using the STEM analysis, and it was found that there was a relationship between circRNA profile 21, miRNA profile 4, and mRNA profile 21. After a comparison of the miRNA predicted in circRNA profile 21 with the miRNA in miRNA profile 4, it was found that the same miRNAs predicting the target genes of this part of miRNAs were also found in their upstream mRNAs; then, the upstream mRNAs were compared with the mRNAs in the middle of mRNA profile 21 to determine the common mRNAs. Finally, mRNAs were associated with miRNA profile 4 and circRNA profile 21 to obtain the ceRNA regulation network, as shown in Figure 4B (Supplemental Table S10).

### 3.4. RT-qPCR Quantification of circRNAs

**Figure 5:**
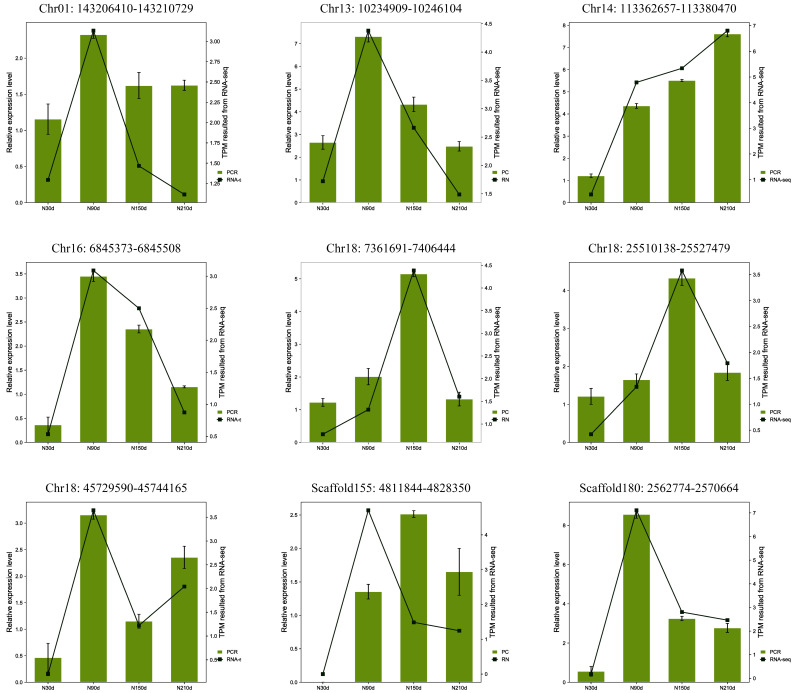
Transcription patterns of Chr18_45729590_45744165, Scaffold180_2562774_2570664, Chr01_143206410_143210729, Chr18_7361691_7406444, Chr18_25510138_25527479, Chr16_6840714_6845508, Chr14_113362657_113380470, and Chr13_10234909_10246104, compared with expression patterns in the RNA-Seq.

Nine circRNAs—namely, Chr18_45729590_45744165, Scaffold180_2562774_2570664, Scaffold155_4811844_4828350, Chr01_143206410_143210729, Chr18_7361691_7406444, Chr18_25510138_25527479, Chr16_6840714_6845508, Chr14_113362657_113380470, and Chr13_10234909_10246104—were randomly selected from the novel circRNAs and quantified by RT-qPCR at four development stages (30, 90, 150, and 210 days after birth). The results showed a concordance between the RNA-Seq and RT-qPCR data, suggesting that the RNA-Seq data were reliable (Figure 5 and Supplemental Table S11).

## 4. Discussion

With advances in sequencing technology, an increasing number of previously obscured small molecules of genetic material are known, including lncRNAs, circRNAs, and exosomes. These small molecules play critical roles in regulating biological growth and development or cell differentiation [16]. As a substance with a stable structure and one that can regulate biological transcriptome in organisms, the circRNA has been confirmed in many organisms such as mice [17], nematodes [18], and drosophila [19]. As an important agricultural economic animal in the world, pigs are widely accepted because of their fast growth, high yield, and good meat quality. Today, extensive research has been performed on pigs. For example, Tang analyzed and identified the circRNAs of a widely used biomedical model animal and found 149 circRNAs related to muscle growth and, at the same time, found that their host genes were significantly involved in muscle development and contraction [20]; moreover, this set of genes was markedly enriched in genes involved in tight junctions and the calcium signaling pathway. They also constructed a first public S. scrofa circRNA database. Morten et al. [21] revealed the highly complex regulatory model of circRNAs by an unbiased analysis of circRNAs in six time points and five brain tissues during porcine embryonic development. An unbiased analysis reveals a high complex regulation pattern of thousands of circRNAs, with a distinct spatiotemporal expression profile, suggesting that circRNA plays an important role in pig brain development. With a sequencing study and comparison of pig ovaries, Xu [22] found that circRNA would affect the litter size of pigs. These studies suggest that we have conducted an increasing level of research on circRNA in pigs. At the same time, circRNA is a molecule with spatiotemporal expression regulation. Therefore, research on circRNAs must be inseparable from time-division research. As a famous pig breed in China, the Ningxiang pig is famous for its high-quality meat quality. When it comes to good meat quality traits, it is inseparable from its body fat content, but so far, the regulatory mechanism of its high-fat content is still not particularly clear. The sources of lipids in pigs can be divided into exogenous and endogenous; exogenous lipids are mainly taken from food, while endogenous lipids are synthesized in the liver; after the exogenous lipid is absorbed into the blood in the small intestine, it forms chyle particles and can decompose the triglyceride (TG) in its core into free fatty acids and glycerol under the action of lipoprotein lipase (LPL) for oxidation and energy supply of extrahepatic tissues; after endogenous lipids are synthesized in the liver, they form very low density lipoprotein (VLDL), which is transported in the blood and can also be decomposed into free fatty acids and glycerol by LPL. LPL is a classical lipid metabolism enzyme, which can catalyze the decomposition of triglycerides in TG rich lipoprotein, chyle particles and VLDL core into fatty acids and monoglycerides for tissue oxidation and energy supply. It plays an important regulatory role in systemic glucose and lipid metabolism [23]. In order to understand the relationship between the mechanisms of endogenous lipid metabolism, fat deposition and transport and circRNA in Ningxiang pigs, we selected the liver tissues of Ningxiang pigs at four different time periods (30d, 90d, 150d, and 210d after birth) for RNA sequencing analysis and compared them with the reference genomes of other pig species. The mapping rate of each sample exceeded 92%, and the percentage of Q30 bases were more than 95% (Table 1). This shows that our sequencing data are reliable and can be used for further research. 11,380, 241,112, 12,904, and 12,943 circRNAs were identified at different stages of liver development, indicating that circRNAs play a time-dependent regulatory role in liver development. At the same time, we found that the amount of circRNAs at 90d was the largest. At the same time, we also analyzed the distribution of circRNA on chromosomes and found that circRNA was mainly distributed on chromosomes 1, 6, and 13 of pigs. According to the research of Tang, most circRNAs in pigs contain multiple exons, and a small part of circRNAs contain introns. The data of this study show that most of the circRNAs identified (68.8%) were exons, followed by intergenic (17.8%) circRNAs, and only a small portion (13.4%) were located in introns; the largest circRNAs length was more than 3000 bp. Kocks Christine [24] found that a large circRNA CDR1AS (containing about 1500 nucleotides) has up to 70 binding sites for miR-7, suggesting that the longer the length of circRNA, the more binding sites it can provide to miRNA.

By analyzing the circRNA obtained in each stage of the liver, it was found that a total of 3705 circRNAs existed in each stage of liver development, indicating that the growth and development of Ningxiang pig are inseparable from the regulation of circRNAs. Through the enrichment analysis of these 3705 circRNAs, the results showed that circRNAs were mainly significantly enriched in the pathways such as complex and coagulation cascades, ECM receiver interaction, tryptophan metabolism, and amoebiasis.

Fat deposition is the main way of energy storage; the amount of fat deposition in animals is a balance between fat anabolism and catabolism, and a significant correlation has been found between liver fat content and body fat content [25]. However, the role of the circRNAs in the liver and how they affect fat deposition and development in animals is still unclear. We identified a large number of circRNAs such as Scaffold180_2562774_2570664, Scaffold155_4811844_ 4828350, Chr18_45729590_45744165, which were found to have high expression in the middle of the liver. Fructose 2,6-bisphosphate (Fru-2,6-P2) is an important metabolite that controls glycolytic and gluconeogenic pathways in several cell types. Its synthesis and degradation are catalyzed by the bifunctional enzyme 6-phosphofructo-2-kinase/fructose 2,6-bisphosphatase (PFK-2). Four genes, designated as 6-phosphofructo-2-kinase/fructose-2,6-bisphosphatase (PFKFB1-4), codify the different PFK-2 isozymes. Studies have shown that high levels of Fru-2,6-P2 in the liver of transgenic mice can lead to accumulation of lipids in periportal cells, and weight gain [26]. During liver development, it was observed that Scaffold180562774_2570664, produced by host gene PFKFB1, was abundantly expressed at 90d. Integrin alpha-9 (ITGA9) [27] can be located in the sphingolipid pathway. Sphingolipids can produce ceramide under the catalysis of sheath phospholipase, and the increase in ceramide leads to fat deposition. At the same time, VLDL and LDL in the process of fat formation are rich in sheath phospholipids. Interestingly, we observed a peak expression of Scaffold155_4811844_4828350 transcribed by ITGA9 as host gene in the developing 90d pig liver. In addition, 3-hydroxyisobutyrate dehydrogenase (HIBADH) is an important part of lipid metabolism pathway [28]. In our study, we found that Chr18_45729590_45744165 host gene is HIBADH, and it had a high expression during Ningxiang pig liver development, and the expression was the highest at 90d. This finding suggests that many circRNAs are highly expressed in varying degrees during liver development, and they will affect the fat metabolism and deposition of Ningxiang pigs.

STEM analysis in this study revealed that all the identified circRNA were classified into five cluster profiles in the liver, containing seven enriched model profiles. Profiles 9 and 10 belonged to the same cluster, which was enriched for intramembrane lipid transporter activity, bile acid biosynthetic process, ATPase-coupled intramembrane lipid transporter activity, and phospholipid binding. In the profiles 21, many circRNAs were enriched for phosphonate and phosphinate metabolism, metabolic pathways, glyoxylate and dicarboxylate metabolism, tryptophan metabolism, propanoate metabolism, and steroid biosynthesis. These enriched pathways are basically related to fat metabolism. For example, the host gene Farnesyl-Diphosphate Farnesyltransferase 1 (FDFT1) of circRNA in circRNA profile 4 was found enriched in the steroid biosynthesis pathway, in which the encoded protein is the first specific enzyme in cholesterol biosynthesis, catalyzing the dimerization of two molecules of farnesyl diphosphate in a two-step reaction to form squalene synthase (SQS), and SQS is a key regulator in cholesterol synthesis [29]. Host gene Choline Phosphotransferase 1 (CHPT1) of circRNA in circRNA profile 21 was found to be a protein-coding gene with related pathways to glycerophospholipid biosynthesis; Gene Ontology (GO) annotations related to this gene include diacylglycerol binding and diacylglycerol choline phosphotransferase activity [30]. According to STEM research results, it is suggested that circRNAs participate in a variety of fatty acid metabolism pathways during the development of Ningxiang pigs.

Competing endogenous RNAs(ceRNA) are widely present in the liver. It is well known that the growth, development, and differentiation of adipocytes in living organisms is an extremely complex process, which is regulated by several transcription factors and genes. However, as the research on adipocytes deepened and the science advanced, scientists found that, in addition to adipose tissue, the liver has an important role in regulating the growth and development of adipocytes. Since adipose tissue in pigs does not synthesize unsaturated fatty acids, the source of unsaturated fatty acids for adipocyte development can only be synthesized by the liver. This process is inevitably regulated by circRNAs. For example, in our study, we found that Chr18_45729590_45744165, produced by HIBADH as a host gene, was highly expressed in liver tissues (Figure 2E and Supplemental Table S6). Combined with the joint analysis results of SETM and ceRNA, it was found that among the predicted target miRNAs of Chr18_45729590_45744165, only ssc-miRNA-378 and ssc-miRNA-378p were significant in the STEM analysis of miRNA. Among the target mRNAs of ssc-miRNA-378 and ssc-miRNA-378p, there are four mRNAs (C7, AR, SLC17A2, and MSTRG.20892.1) that are significant in mRNA STEM analysis (Figure 4B and Supplemental Table S10). SLC17A2 is associated with diseases such as nephrolithiasis/osteoporosis, hypophosphatemia 1, and Fanconi renotubular syndrome 2 (http://www.genecards.org accessed on 15 January 2021). Studies have shown that the increase in adipose tissue mass is usually related to the pro-inflammatory state. At the same time, the cross-sectional data of the Framingham Heart Study show that the volume of subcutaneous adipose tissue is related to the increase in the level of pro-inflammatory cytokine C-reactive protein [31,32]. Complement component C7 (C7) is a mosaic protein composed of 821 amino acids. The third carboxyl-terminal contains four cysteine-rich fragments. All cysteines exist in small units of 35–77 amino acids. These amino acids have homology with low-density lipoprotein receptors. In our study, the complement C7 gene was found to be associated with miRNA in stem analysis [33,34]. The Androgen receptor (AR) is the mediator of androgen action. At present, the AR element sequence motif of the AR binding site is considered to be ACATTTGT in the LKB1 gene promoter [35]. Liver kinase B1 (LKB1) is the main upstream regulator of the AMPK– ACC (acetyl-CoA carboxylase) pathway, and the AMPK–ACC pathway plays an important role in the process of hepatic triglyceride storage [36]. Through this study, we found that circRNA: Chr18_45729590_45744165 in liver tissue will regulate genes related to the development, transportation, and deposition of adipose tissue through the “miRNA sponge” during the growth and development of Ningxiang pigs.

## 5. Conclusions

This study revealed the expression profiles of circRNAs in Ningxiang pig liver at different periods of development and determined that there is differential coexpression. Through enrichment analysis of these circRNAs, it was revealed that host genes were involved in metabolism-related signaling pathways and fatty acid anabolism. Through STEM analysis, many circRNAs involved in fat metabolism, transport, and deposition pathways were screened, and the first circRNA–miRNA–mRNA regulation network map in Ningxiang pig liver was constructed. The highly expressed circRNAs related to fat deposition were verified and were consistent with RNA-Seq results. The results of this study provide a significant expression database for studying circRNAs during pig fat deposition.

## Figures and Tables

**Table 1:** Sequencing basic data of four different developmental stages of Ningxiang pig liver.

Terms	NX30d	NX90d	NX150d	NX210d
Raw reads number	56500489	55531984	45030983	51897636
Clean reads number	55574608	54755520	44381475	51069717
Clean reads rate	98.36%	98.60%	98.56%	98.40%
Clean Q30 bases rate	95.14%	95.55%	95.28%	95.52%
Mapped reads	102827570	103499271	83368071	95480783
Mapping rate	92.51%	94.51%	93.92%	93.48

The values represent the reads and proportion that were compared with those in the Ningxiang pig reference genome (PRJNA531381) using the Hisat2 program.

## Data Availability

The dataset generated and analyzed in this study can be obtained in the repository, as follows: Whole transcriptome high-throughput sequencing data access code: PRJNA721288 (https://www.ncbi.nlm.nih.gov/bioproject/PRJNA721288/, accessed on 12 January 2021).

## Supplementary Materials

The following supporting information can be downloaded at: https://www.mdpi.com/article/10.3390/genes13050746/s1, Table S1: List of identified circRNAs during the development of the liver, Table S2: circRNA length distribution and exon, intron, intergenic distribution in liver, Table S3: List of differentially expressed (DE) circRNAs in liver, Table S4: List of common differentially expressed (DE) circRNAs for three developmental stages (90d, 150d, and 210d) vs. 30d in liver, Table S5: KEGG pathways enrich of circRNAs between two closed time points in liver tissue, Table S6: List of top 20 circRNAs expressed in liver, Table S7: STEM analysis of dynamic expression patterns across the four development stages, Table S8: KEGG pathways enrich of circRNAs list in model profiles 4 of STEM, Table S9: The ceRNA networks of circRNAs and their targeted miRNAs and mRNAs, Table S10: The ceRNA networks of mRNA profile 21, miRNA profile 4 and circRNA profile 21, Table S11: Primer.

## References

[1] Yang F., Gao H., Zhang Y., Liao Y., Zeng Q., He X., Xu K., He J. (2021). Optimizing conditions of electronic nose for rapid detection of flavor substances in Ningxiang Pork. J. Food Process Eng..

[2] Ma H., Jiang J., He J., Huifang L., Han L., Gong Y., Li B., Zonggang Y., Shengguo T., Zhang Y. (2021). Long-read assembly of the Chinese indigenous Ningxiang pig genome and identification of genetic variations in fat metabolism among different breeds. Mol. Ecol. Resour..

[3] He Q., Ren P., Kong X., Wu Y., Wu G., Li P., Hao F., Tang H., Blachier F., Yin Y. (2012). Comparison of serum metabolite compositions between obese and lean growing pigs using an NMR-based metabonomic approach. J. Nutr. Biochem..

[4] Zhang K., Tao C., Xu J., Ruan J., Xia J., Zhu W., Xin L., Ye H., Xie N., Xia B. (2021). CD8 T Cells Involved in Metabolic Inflammation in Visceral Adipose Tissue and Liver of Transgenic Pigs. Front. Immunol..

[5] Ahn J., Lee H., Chung C., Ha T. (2011). High fat diet induced downregulation of microRNA-467b increased lipoprotein lipase in hepatic steatosis. Biochem. Biophys. Res. Commun..

[6] Smolka C., Schlösser D., Hohnloser C., Bemtgen X., Jänich C., Schneider L., Martin J., Pfeifer D., Moser M., Hasselblatt P. (2021). MiR-100 overexpression attenuates high fat diet induced weight gain, liver steatosis, hypertriglyceridemia and development of metabolic syndrome in mice. Mol. Med..

[7] Wang J., Ren Q., Hua L., Chen J., Zhang J., Bai H., Li H., Xu B., Shi Z., Cao H. (2019). Comprehensive Analysis of Differentially Expressed mRNA, lncRNA and circRNA and Their ceRNA Networks in the Longissimus Dorsi Muscle of Two Different Pig Breeds. Int. J. Mol. Sci..

[8] Huang R., Zhang Y., Han B., Bai Y., Zhou R., Gan G., Chao J., Hu G., Yao H. (2017). Circular RNA HIPK2 regulates astrocyte activation via cooperation of autophagy and ER stress by targeting MIR124-2HG. Autophagy.

[9] Zang J., Lu D., Xu A. (2020). The interaction of circRNAs and RNA binding proteins: An important part of circRNA maintenance and function. J. Neurosci. Res..

[10] Yang W., Zhao J., Zhao Y., Li W., Zhao L., Ren Y., Ou R., Xu Y. (2020). Hsa_circ_0048179 attenuates free fatty acid-induced steatosis via hsa_circ_0048179/miR-188-3p/GPX4 signaling. Aging.

[11] Yu G., Yang Z., Peng T., Lv Y. (2021). Circular RNAs: Rising stars in lipid metabolism and lipid disorders. J. Cell. Physiol..

[12] Li B., Yang J., He J., Gong Y., Xiao Y., Zeng Q., Xu K., Duan Y., He J., Ma H. (2021). Spatiotemporal Regulation and Functional Analysis of Circular RNAs in Skeletal Muscle and Subcutaneous Fat during Pig Growth. Biology.

[13] Han P., Li P., Zhou W., Fan L., Wang B., Liu H., Gao C., Du T., Pu G., Wu C. (2020). Effects of various levels of dietary fiber on carcass traits, meat quality and myosin heavy chain I, IIa, IIx and IIb expression in muscles in Erhualian and Large White pigs. Meat Sci..

[14] Ernst J., Bar-Joseph Z. (2006). STEM: A tool for the analysis of short time series gene expression data. BMC Bioinform..

[15] Liu T., Zhou L., He Z., Chen Y., Jiang X., Xu J., Jiang J. (2021). Circular RNA hsa_circ_0006117 Facilitates Pancreatic Cancer Progression by Regulating the miR-96-5p/KRAS/MAPK Signaling Pathway. J. Oncol..

[16] Gong Y., Zhang Y., Li B., Xiao Y., Zeng Q., Xu K., Duan Y., He J., Ma H. (2021). Insight into Liver lncRNA and mRNA Profiling at Four Developmental Stages in Ningxiang Pig. Biology.

[17] Huang Y., Ge W., Ding Y., Zhang L., Zhou J., Kong Y., Cui B., Gao B., Qian X., Wang W. (2021). The circular RNA circSLC7A11 functions as a mir-330-3p sponge to accelerate hepatocellular carcinoma progression by regulating cyclin-dependent kinase 1 expression. Cancer Cell Int..

[18] Kim E., Kim Y.K., Lee S.-J.V. (2021). Emerging functions of circular RNA in aging. Trends Genet..

[19] Krishnamoorthy A., Kadener S. (2021). Using Drosophila to uncover molecular and physiological functions of circRNAs. Methods.

[20] Liang G., Yang Y., Niu G., Tang Z., Li K. (2017). Genome-wide profiling of Sus scrofa circular RNAs across nine organs and three developmental stages. DNA Res..

[21] Venø M.T., Hansen T.B., Venø S.T., Clausen B.H., Grebing M., Finsen B., Holm I.E., Kjems J. (2015). Spatio-temporal regulation of circular RNA expression during porcine embryonic brain development. Genome Biol..

[22] Xu G., Zhang H., Li X., Hu J., Yang G., Sun S. (2019). Genome-Wide Differential Expression Profiling of Ovarian circRNAs Associated With Litter Size in Pigs. Front. Genet..

[23] Kersten S. (2021). Role and mechanism of action of angiopoietin-like protein ANGPTL4 in plasma lipid metabolism. J. Lipid Res..

[24] Kocks C., Boltengagen A., Piwecka M., Rybak-Wolf A., Rajewsky N. (2018). Single-Molecule Fluorescence In Situ Hybridization (FISH) of Circular RNA CDR1as. Methods Mol. Biol..

[25] Ramírez-Vélez R., Izquierdo M., Correa-Bautista J., Correa-Rodríguez M., Schmidt-RioValle J., González-Jiménez E., González-Jiménez K. (2018). Liver Fat Content and Body Fat Distribution in Youths with Excess Adiposity. J. Clin. Med..

[26] Duran J., Navarro-Sabate A., Pujol A., Perales J.C., Manzano A., Obach M., Gómez M., Bartrons R. (2008). Overexpression of ubiquitous 6-phosphofructo-2-kinase in the liver of transgenic mice results in weight gain. Biochem. Biophys. Res. Commun..

[27] Demirkan A., van Duijn C.M., Ugocsai P., Isaacs A., Pramstaller P.P., Liebisch G., Wilson J.F., Johansson Å., Rudan I., Aulchenko Y.S. (2012). Genome-wide association study identifies novel loci associated with circulating phospho- and sphingolipid concentrations. PLoS Genet..

[28] Nilsen M.S., Jersin R.Å., Ulvik A., Madsen A., McCann A., Svensson P.-A., Svensson M.K., Nedrebø B.G., Gudbrandsen O.A., Tell G.S. (2020). 3-Hydroxyisobutyrate, A Strong Marker of Insulin Resistance in Type 2 Diabetes and Obesity That Modulates White and Brown Adipocyte Metabolism. Diabetes.

[29] Dong X., Zhu Y., Wang S., Luo Y., Lu S., Nan F., Sun G., Sun X. (2020). Bavachinin inhibits cholesterol synthesis enzyme FDFT1 expression via AKT/mTOR/SREBP-2 pathway. Int. Immunopharmacol..

[30] Cadenas C., Vosbeck S., Hein E.-M., Hellwig B., Langer A., Hayen H., Franckenstein D., Büttner B., Hammad S., Marchan R. (2012). Glycerophospholipid profile in oncogene-induced senescence. Biochim. Biophys. Acta.

[31] Yudkin J.S., Stehouwer C.D., Emeis J.J., Coppack S.W. (1999). C-reactive protein in healthy subjects: Associations with obesity, insulin resistance, and endothelial dysfunction: A potential role for cytokines originating from adipose tissue?. Arterioscler. Thromb. Vasc. Biol..

[32] Pou K.M., Massaro J.M., Hoffmann U., Vasan R.S., Maurovich-Horvat P., Larson M.G., Keaney J.F., Meigs J.B., Lipinska I., Kathiresan S. (2007). Visceral and subcutaneous adipose tissue volumes are cross-sectionally related to markers of inflammation and oxidative stress: The Framingham Heart Study. Circulation.

[33] DiScipio R.G., Chakravarti D.N., Muller-Eberhard H.J., Fey G.H. (1988). The structure of human complement component C7 and the C5b-7 complex. J. Biol. Chem..

[34] Van der Meer B.W., Fugate R.D., Sims P.J. (1989). Complement proteins C5b-9 induce transbilayer migration of membrane phospholipids. Biophys. J..

[35] Heo J., Lee S., Jo S., Ko J., Kwon H., Hong E. (2021). Hepatic LKB1 Reduces the Progression of Non-Alcoholic Fatty Liver Disease via Genomic Androgen Receptor Signaling. Int. J. Mol. Sci..

[36] Imai K., Inukai K., Ikegami Y., Awata T., Katayama S. (2006). LKB1, an upstream AMPK kinase, regulates glucose and lipid metabolism in cultured liver and muscle cells. Biochem. Biophys. Res. Commun..

